# Effects of hydroxyapatite-coated porous titanium scaffolds functionalized by exosomes on the regeneration and repair of irregular bone

**DOI:** 10.3389/fbioe.2023.1283811

**Published:** 2023-10-31

**Authors:** Hanyu Shao, Qiyue Zhang, Mingman Sun, Ming Wu, Xu Sun, Qiang Wang, Shuang Tong

**Affiliations:** ^1^ Department of Plastic Surgery, First Hospital of China Medical University, Shenyang, China; ^2^ Liaoning Provincial Key Laboratory of Oral Diseases, School and Hospital of Stomatology, China Medical University, Shenyang, China

**Keywords:** adipose-derived stem cells, exosomes, bone tissue engineering, porous titanium alloy scaffold, hydroxyapatite

## Abstract

As a traditional bone implant material, titanium (Ti) and its alloys have the disadvantages of lack of biological activity and susceptibility to stress shielding effect. Adipose stem cells (ADSCs) and exosomes were combined with the scaffold material in the current work to effectively create a hydroxyapatite (HA) coated porous titanium alloy scaffold that can load ADSCs and release exosomes over time. The composite made up for the drawbacks of traditional titanium alloy materials with higher mechanical characteristics and a quicker rate of osseointegration. Exosomes (Exos) are capable of promoting the development of ADSCs in porous titanium alloy scaffolds with HA coating, based on experimental findings from *in vitro* and *in vivo* research. Additionally, compared to pure Ti implants, the HA scaffolds loaded with adipose stem cell exosomes demonstrated improved bone regeneration capability and bone integration ability. It offers a theoretical foundation for the combined use of stem cell treatment and bone tissue engineering, as well as a design concept for the creation and use of novel clinical bone defect repair materials.

## 1 Introduction

New histocompatibility scaffold materials are being developed in bone tissue engineering research to use these materials as templates for bone tissue regeneration to support loading, cell adhesion, and tissue defect repair. The creation of a novel artificial bone graft replacement material with strong mechanical properties as well as favourable bone healing capabilities may promote bone regeneration in clinical circumstances (Mandrycky et al., 2016; Deng et al., 2021). At present, the artificial hard tissue repair materials available in clinics and the medical materials market can be broadly divided into two categories (Jia et al., 2023). Calcium-based materials, like HA and β-tricalcium phosphate, are beneficial for their ability to provide strong bone guidance and degradation capabilities (Jia et al., 2021). Due to their inferior mechanical properties, brittleness, and limited ability to bear weight, using them alone to reconstruct substantial bone defects is not an optimal solution (Fan et al., 2022). Metal-based materials, including stainless steel, cobalt alloys and titanium alloys, possess exceptional mechanical strength, hardness, toughness, impact resistance, and fatigue resistance. Due to these properties, they are suitable for reconstructing hard tissues in weight-bearing areas. However, they cannot induce bone growth (Chia and Wu, 2015a; Qi et al., 2020; Cresnar et al., 2021; Huang et al., 2021). Some researchers have attempted to use porous metal materials to meet the requirements of bone-filling repair and mechanical stability. Nevertheless, the inert characteristics of pure metal-based materials hinder bone growth (Civantos et al., 2019; Ma et al., 2020).

HA has been utilized in clinical practice for numerous years due to its similar structural composition to bone tissue and its satisfactory biocompatibility (Bordea et al., 2020; Duan et al., 2020; Huang et al., 2022). Moreover, HA exhibits positive bone conductivity, which promotes the adhesion and development of bone tissue (He et al., 2023). It creates chemical bonds with bone tissue, thus improving the integration between the material and bone tissue. Due to its poor mechanical strength and high brittleness, it is unsuitable for repairing bone defects at weight-bearing sites. Conversely, HA is an excellent coating material (Lu et al., 2019) that can prevent the release of harmful metal ions into surrounding bone tissue, which can prevent osteoporosis, neurological disorders and other diseases (Jafarian et al., 2008).

In recent years, titanium and its alloys have been extensively investigated, used and developed owing to their excellent physical and chemical properties when compared to other metals (Chia and Wu, 2015b). Nevertheless, these materials still have some limitations in clinical applications (Al-Tamimi et al., 2017). The high mechanical strength of titanium and its alloys can cause stress shielding after implantation, which may lead to the absorption of surrounding bone tissue, loosening or fracture of the prosthesis (Al-Tamimi et al., 2017), resulting in low binding strength and failure of tissue reconstruction and repair. Porosity can affect the overall density, strength and elastic modulus of metal implants (Doi et al., 2020). Adjusting pore size and porosity can effectively reduce or eliminate the stress-shielding effect and promote the adhesion, proliferation and differentiation of osteoblasts (Wang et al., 2022b), leading to the growth of new bone tissue into the pores and forming a stable structure with the implant (Wang et al., 2022a). Pores larger than 100 μm can facilitate cell and tissue development, along with vascularization and nutrient transport (Al-Tamimi et al., 2017). Meanwhile, pores that are sized between 150 and 1,000 μm are most conducive to the growth of mineralized bone tissue (Koizumi et al., 2019). The growth of bone in porous materials is directly proportional to the size of the pores. However, larger pore sizes do not necessarily promote bone tissue growth. In fact, materials made of porous titanium with pore sizes of 400–600 μm have been found to promote bone formation at a higher rate than those with pore sizes of 1,000 μm (Liu et al., 2022). Additionally, pores facilitated the free transmission of body fluids, nutrient supply, metabolic waste excretion, tissue regeneration and reconstruction, and accelerate the whole repair process (Zhang et al., 2020b).

Bone tissue repair involves a series of actions by stem cells, such as proliferation, differentiation, recognition of extracellular matrix and signalling molecules, expression of related factors and targeting (Li et al., 2018). Exosomes, which are extracellular vesicles, have been found to play a significant role in repair and regeneration. Exosomes are small vesicles, ranging from 50 to 120 nm, that can be secreted by various types of cells such as dendritic cells, mast cells, epithelial cells, and stem cells. These vesicles are created in endosomal compartments and are released into the extracellular environment by fusing with the plasma membrane, which enables intercellular communication and exchange of information (Meng et al., 2022). Experimental studies have demonstrated the pro-regenerative role of exosomes in other tissues and organs, including the heart, lungs, kidneys, and brain (Bjorge et al., 2017). Besides, recent studies have demonstrated that exosomes can promote the differentiation of mesenchymal stem cells into osteoblasts (Staruschenko et al., 2006; Li et al., 2021; Meng et al., 2022). Lee et al. (2023) demonstrated that exosomes can be efficiently fixed to the hydroxyapatite surface by binding to hydroxyapatite coated 3D bone implants with extended localization capabilities for implantable applications. Exosomes activate and participate in different intracellular pathways to promote bone formation through transporting cargo during differentiation of bone marrow mesenchymal stem cells (Wong et al., 2020). And they can promote the formation of new bone and blood vessels by activating the BMP-2/Samd1/RUNX2 signalling pathway (Zhang et al., 2020a). In addition, studies have also demonstrated that exosomes can promote bone formation by up-regulating miRNA and activating the MAPK/PI3K/Akt signalling pathway (Zhai et al., 2020). ADSCs are similar to bone marrow-derived mesenchymal stem cells, and can maintain self-renewal and have multidirectional differentiation potential (Shukla et al., 2020; Li et al., 2022). Therefore, it is believed that ADSCs’ exosomes can be used to promote bone regeneration and repair and can be incorporated into tissue-engineered bone implants.

When dealing with large bone defect areas, neither stem cells nor inducible factors can act alone. Instead, a scaffold material must be used as a carrier to provide a transitional place for stem cells to induce differentiation *in vivo.* By modifying the surface of titanium and its alloy can be preserved while also meeting clinical requirements for bone repair. Nano-hydroxyapatite (n-HA) is particularly effective at promoting cell proliferation and differentiation due to its strong adsorption capacity. Implanting seed cells immediately after three-dimensional scaffolds with composite coating can reduce the risk of contamination and uncertainty during the procedure (Cao et al., 2022). This procedure is called *in vivo* construction and has been recognized by the academic community. Therefore, HA-coated porous titanium scaffolds capable of carrying ADSCs and releasing exosomes can be prepared *in vivo* to construct tissue-engineered bone with better mechanical properties. Selective laser melting (SLM) is a 3D-printing method that allows for the precise control of pore parameters and the creation of complex-shaped porous metal materials (Tian et al., 2019; Attarilar et al., 2021; Liu et al., 2021; Yi et al., 2022a; Liu et al., 2022). Micro-arc oxidation (MAO), also known as plasma electrolytic oxidation (PEO) (Carobolante et al., 2020; Rokosz et al., 2020), can be used to coat the surface of titanium alloys with HA (Bartolomeu et al., 2021). The MAO surface modification promoted the adhesion and growth of bone tissue owing to the rough porous surface (Xu et al., 2012).

A new composite material was created using SLM and MAO technology to address the limitation of traditional titanium alloy-based material in repairing bone tissue defects (Zglobicka et al., 2019; Okazaki and Mori, 2021). The effect of composite materials on bone repair and bone integration has been verified through a series of experiments, which not only provides an experimental basis for bone tissue engineering and stem cell therapy, but also makes a new attempt for the development of new bone defect repair materials.

## 2 Materials and methods

### 2.1 Preparation and characterization

The 3D digital models of porous titanium alloy scaffolds with outer dimensions of 8 mm × 4 mm×3 mm and 4 mm × 4 mm×3 mm was prepared using 3D printing technology with the basic structural unit of cubic and pore parameters of 70% porosity and pore diameter of 550 μm. Then the Ti6Al4V powder (powder size less than 50 μm) was preheated using an electron beam in a vacuum environment (10^−4^–10^−5^ mbar). Set the laser spot diameter to 70 μm, laser power to 260 W, and scanning speed to 1,200 mm/s using SLM devices (3D Systems, United States, model: Prox DMP200) melted Ti6Al4V powder material layer by layer at a scanning speed of 400 mm/s to obtain porous Ti6Al4V support material. The sample is soaked in a dilute hydrofluoric acid solution with a concentration of 40% for 1 min to remove the unmelted metal powder. Then use sandpaper to sand and polish the material. The detail can be found in our previous study (Yi et al., 2022a).

Porous Ti6Al4V scaffolds were prepared using SLM technology and placed into the required electrolyte (The main components of the electrolyte: β-disodium glycerophosphate 0.01–0.03 mol/L, calcium acetate 0.1–0.3 mol/L, zinc acetate 0.01–0.04 mol/L and the conductivity of less than 1 μS/cm of deionized water). The scaffold was targeted as the anode, and HA (Alfa-Aesar Co., Ward HILL, MA, United States) was targeted as the cathode. Oxidation was performed at a constant current (3 A/dm^2^ for 20–30 min). A scanning electron microscope (SSX-550, SHIMADZU, Japan) was used to observe the surface morphology of porous Ti6Al4V scaffolds and HA-coated porous Ti6Al4V (HA-Ti) scaffolds prepared through MAO. An Energy Dispersive X-ray Spectrometer (EDS) (D/max2500, Japan) was used to detect the HA-Ti scaffolds and analyse the surface elements.

Six cube-shaped specimens of 4 mm × 4 mm × 3 mm (length × width × height) were prepared. The HA-Ti scaffolds were immersed in six isopropanol tubes of volume V_1_, and isopropanol was sucked into the wells through negative pressure suction until there were no bubbles. At this point, the total volume of the sample and isopropanol was V_2_, and the volume of isopropanol remaining after the material was removed was V_3_. The total volume of the frame material was calculated as V_2_–V_3_, and the volume of isopropanol in the wells was calculated as V_1_–V_3_. The porosity of the frame was calculated as follows: ε = (V_1_–V_3_)/(V_2_–V_3_) ×100%. Finally, Micro-CT was performed to observe internal structural parameters such as porosity, pore size and trabecular diameter. The data obtained from the two measurement methods were compared.

The compressive strength of porous Ti and HA-Ti scaffolds was tested using a universal mechanical testing machine (ZWICK Z2005, Germany). According to the international standard, the scaffolds were made into a cube of 4 mm × 4 mm × 3 mm (length × width × height). Compressive strength was calculated as follows: P(MPa) = F(N)/S(mm^2^), where P is compressive strength, F is pressure and S is the stress area.

### 2.2 Isolation, culture and identification of ADSCs

ADSCs were subcutaneously derived from aspirated adipose tissue from a 20–30 years old woman without any infectious or systemic diseases, who signed an informed consent form. The study was reviewed by the ethics committee (China Medical University).

Human adipose tissue was obtained under aseptic conditions and digested with 0.25% trypsin (at a ratio of 1:1; Hyclon, United States) and 0.1% collagenase type I (Sigma Chemical Co., St. Louis, MO, United States) at a constant temperature of 37°C on a shaker at 190 r/min for 30 min (TDZ5-WS, China). The digested tissue sample was centrifuged at 1,500 rpm for 10 min at room temperature. The supernatant was discarded, resuspended in high-sugar DMEM (Hyclon, United States) supplemented with 10% FBS (Hyclon, United States), reinoculated in culture bottles and cultured at 37°C and 5% CO_2_ with saturated humidity (Thermo Fisher Scientific, United States).

Third-generation ADSCs with adequate growth were reconfigured into a cell suspension at a concentration of 1 × 10^9^/L, and murine non-specific IgG antibody was added to block the possible Fc receptors. The cells were treated with the corresponding primary antibody (mouse anti-human CD29, CD34, CD44, and CD31 [1:50]) and fluorescent-labelled secondary antibody (sheep anti-mouse IgG-FIFC at a concentration of 1:200) and incubated in a thermostatic water bath (HH-4, China) at 37°C for 30 min. Thereafter, the cells were washed with and resuspended in PBS, and the fluorescence intensity of CD29, CD44, CD34, CD31, and VE-cadherin was detected on a flow cytometer (guava easy Cyte 6-2L, United States). A total of 1 × 10^4^ cells were counted in each tube and analysed using the software.

### 2.3 Extraction and observation of ADSCs-Exos

The fourth generation of well-grown ADSCs were selected and the exosomes released in the undifferentiated stage were collected by overspeed centrifugal method. The dead cells were removed by centrifugation at 4°C and 2000 r/min for 30 min. Centrifuge 2 × 10^4^ r/min for 60 min, filter with 0.22 μm filter, and discard supernatant. After centrifugation at 1 × 10^5^ r/min for 60 min, the supernatant was discarded and the precipitate was re-suspended with PBS after precooling. ADSCs-Exos was purified by centrifugation at 1×10^5^ r/min for 70 min. Exos were observed by transmission electron microscopy (Hitachi, Japan).

### 2.4 *In vitro* study

#### 2.4.1 Co-culturing cells with scaffolds

Because previous studies showed that HA-Ti scaffolds are more efficient in promoting cell proliferation and adhesion than Ti scaffolds (Liang et al., 2022), only the relationship between ADSCs-derived exosomes (ADSCs-Exos) and HA-Ti scaffolds was examined in this study. The prepared scaffolds were divided into the following groups: ADSCs-Exos/HA-Ti, ADSCs-HA-Ti and blank control (ADSCs-Ti) groups. The sterilized material was added to a 96-well plate containing ADSCs at a concentration of 1×10^5^/mL. The plate was then fully cultured at 37°C with 5% CO_2_.

#### 2.4.2 Early cell adhesion

After 1, 3, and 6 h of culture, six samples were collected from each group (at each time point) to make cell suspensions. Cells were counted using a cell counting plate, and the cell adhesion rate was calculated as follows: [(S_1_-S)/S_1_]×100%, where S_1_ is the number of inoculated cells, and S is the number of unattached cells. Scanning electron microscopy was used to observe the growth of cells in the scaffold. Cells were collected on days 1, 3, and 7 of culture. After treatment, cell morphology was observed and photographed on a scanning electron microscope (JSM-TM3000, Japan).

#### 2.4.3 CCK-8 assay

After 1, 3, 5, and 7 days, six samples were collected from each group (at each time point) and incubated with 100 μL of CCK-8 solution (US Everbright Inc., United States) in a CO_2_ incubator for 4 h. Thereafter, 300 μL of the sample was collected from each well and placed in a 96-well culture plate. The absorbance of the sample was measured at 450 nm on a microplate reader (Infinite M200, Tecan, Austria).

#### 2.4.4 Alkaline phosphatase activity

After 1, 4, 7, and 10 days of culture, the activity of alkaline phosphatase (ALP) was evaluated using the Alkaline Phosphatase Assay Kit (Beyotime, China) according to the manufacturer’s instructions. Protein concentration was measured using a BCA protein assay kit (Beyotime, China) according to the manufacturer’s instructions. The activity of ALP in all samples was normalized based on the protein concentration.

### 2.5 *In vivo* experiments

#### 2.5.1 Bone defect rabbit model and grouping

A total of 18 healthy male New Zealand rabbits, about 6 months old and weighing 2–3 kg were used in this study. All animal experiments were performed by the standards recommended in the Guiding Opinions on Treating Experimental Animals by the Ministry of Science and Technology. All rabbits were purchased at least 2 weeks before the experiment and fed a standard daily diet. The animals were used for the experiment after verifying that their diet, activity and mental state were normal.

ADSCs were isolated from the left and right inguinal adipose tissue of rabbits, and ADSCs-Exos were extracted through differential centrifugation. The specific method was the same as that used in the previous part of the study. The rabbits were randomly divided into the following three groups (*n* = 3): Rabbit-ADSCs-Exos/HA-Ti, rabbit-ADSCs-HA-Ti and rabbit-ADSCs-Ti (blank control group).

The rabbits were anesthetized by intramuscular injection of xylazine hydrochloride at a dose of 0.1 mL/kg in gluteus maximus and intravenous injection of 25 wt% urethane solution in normal saline at a dose of 3 mL/kg in auricular vein. A transverse incision about 2 cm long was made under the skin of both the mandible and the neck. The incision was dissected to the bone surface to fully reveal the mandibular lingual mouth and zygomatic bone surface. A full-thickness bone defect of about 8 mm × 4 mm was made at the middle mandibular margin. In the first group, HA-Ti scaffolds incorporated with rabbit-ADSCs-Exos and HA-Ti scaffolds incorporated with rabbit ADSCs were implanted at the bilateral defect sites. In the second group, HA-Ti scaffolds incorporated with rabbit-ADSCs-Exos and blank control group scaffolds were implanted at the bilateral defect sites. In the third group, HA-Ti scaffolds incorporated with rabbit ADSCs and blank control scaffolds were implanted at the bilateral defect sites, and the wounds were tightly sutured. After the operation, the rabbits received a subcutaneous injection of penicillin for 3 days.

#### 2.5.2 General observation

Samples were collected from the three groups, and their morphology was analysed. The integration of the scaffold with surrounding tissue and the adhesion of cells to fibrous granulation tissue was observed. Additionally, proper implantation of the scaffold was ensured.

#### 2.5.3 X-ray and Micro-CT examination

X-ray and Micro-CT examination was performed at 4, 8, and 12 weeks post-operatively to evaluate the position of the scaffold and the repair of the surrounding bone defect area.

#### 2.5.4 The determination of newly mineralized bone volume, bone mineral density, and biomechanical

To assess the location of the implant and the quality and volume of new bone, two rabbits from each group were anesthetized at 4, 8, and 12 weeks postoperatively for Micro-CT observation (Inveon MM Gantry, Germany) of the defect site. Additionally, the repair of bone defects around the implant was evaluated. Bone mineral density was measured through dual-energy X-ray absorptiometry (HologicQDR-2000, China) at 4, 8, and 12 weeks postoperatively. Biomechanical properties were assessed using a universal mechanical testing machine (ZWICK Z2005, Germany) at 4, 8, and 12 weeks postoperatively.

### 2.6 Statistical analysis

The measurement data were expressed as mean ± standard deviation. The SPSS Statistics (version 19.0) software was used to perform t-tests. *p* < 0.05 indicates that the difference is statistically significant.

## 3 Results

### 3.1 Characterisation of HA-Ti

**FIGURE 1:**
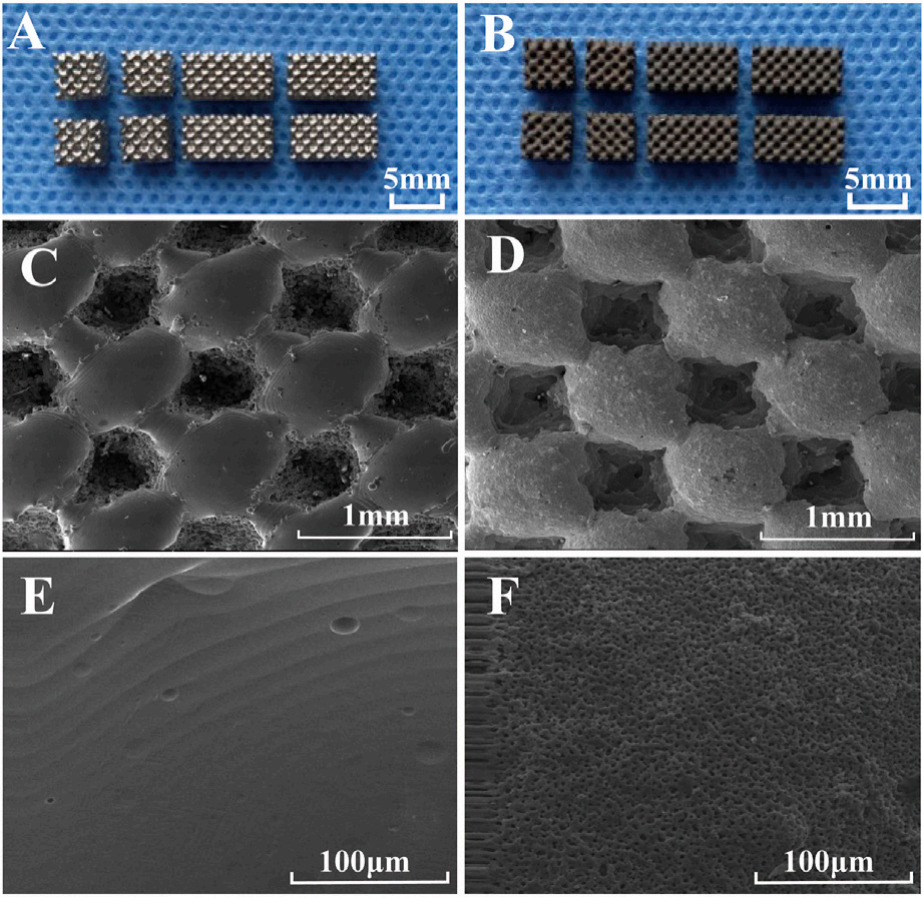
Morphological characterization of scaffolds: General observation: **(A)** porous scaffold and **(B)** HA-coated porous scaffold. SEM microstructure: **(C,E)** porous scaffold and **(D,F)** HA-coated porous scaffold.

The external morphology and size of the two types of porous titanium alloy scaffolds prepared using SLM technology are shown in Figure 1. Porous titanium alloy scaffolds had a smooth surface and regular pore morphology (Figure 1A). Porous titanium alloy scaffolds with HA composite coating had a black-grey rough surface and no metallic luster, and the coating was tightly bound without shedding (Figure 1B). Figures 1C–F demonstrate the surface morphology of uncoated and HA-coated porous titanium alloy scaffolds (prepared via MAO) observed via scanning electron microscopy (SEM). SEM (Figures 1C, E) revealed that titanium alloy scaffolds had a smooth surface and regular shape consistent with the design, whereas HA-coated porous titanium alloy scaffolds (Figures 1D, F) had a rough surface, good continuity and certain pores on the coating surface without any cracks or peeling. The mechanical test results show that the compressive strength of porous titanium alloy is 92 ± 4 MPa and the elastic modulus is 3.2 ± 0.3 GPa.

The porosity of the porous titanium alloy scaffold measured by the negative pressure suction method is 69% ± 5%. While, the Micro-CT showed the porosity of porous titanium alloy scaffolds was 69% ± 3%, the diameter of trabecular metal was 244 ± 21 μm and the pore size was 542 ± 26 μm. The EDS result showed that the surface of the implanted rod mainly contained active elements such as Ca, P, and O (from HA coating). On calculating the atomic percentage of calcium and phosphorus, the ratio of the two elements was found to be approximately 1.67 (Table 1).

**TABLE1:** The weight percentage and atomic percentage of each element on the surface of the HA-Ti scaffold.

Element	C	O	Na	Al	Si	P	Ca	Ti	V
wt%	11.9	56.4	1.2	1.7	0.6	0.4	7.0	17.6	0.5
at%	18.8	66.4	1.0	1.2	0.4	1.9	3.3	6.9	0.2

### 3.2 Results of *in vitro* experiments

#### 3.2.1 Morphological characteristics and identification of ADSCs and observation of exos

**FIGURE 2:**
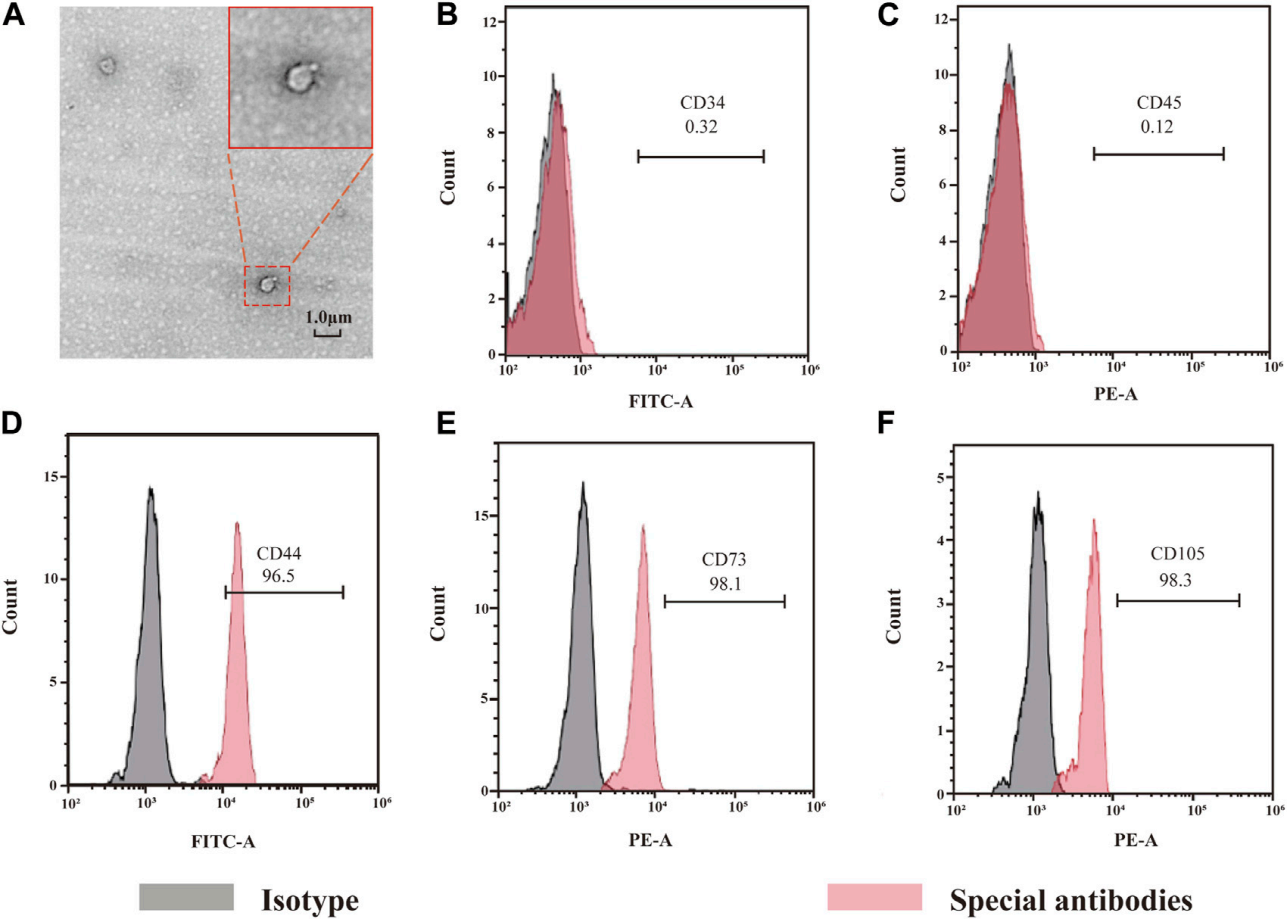
Morphology of exosome and identification of ADSCs. Picture A shows the morphology of exosomes under SEM, showing a double-layer membrane structure. Pictures from B to D show the results of ADSCs detection by flow cytometry.

**FIGURE 3:**
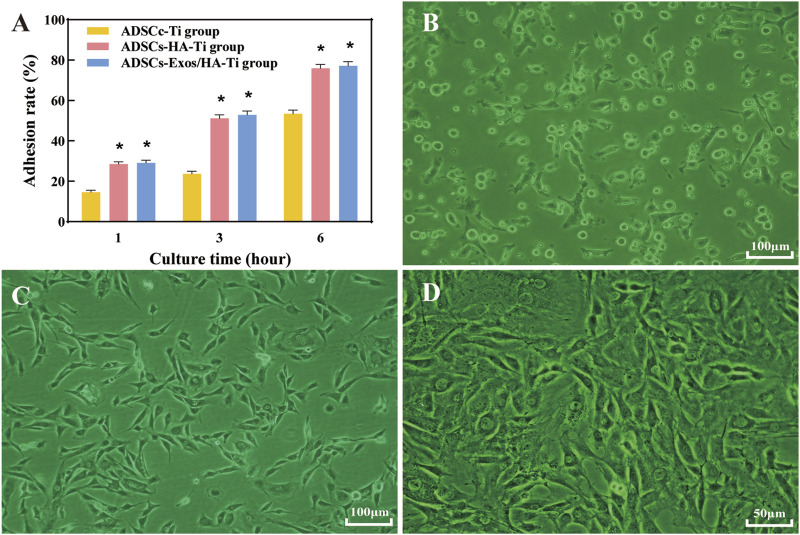
Cell adhesion: Cell adhesion rate after culturing for 1, 3 and 6 h in different groups **(A)**; Cell adhesion graphics after culturing without scaffolds for 1 day **(B)**, 3 **(C)** and 12 days **(D)** in different groups. (*p < 0.05)

For approximately 1 h of culture, ADSCs adhered to the wall. For 24 h, ADSCs had a long-spindle or polygonal shape, with round nuclei, and were mixed with leukocytes and lymphocytes (Figure 3B). For 3 days, a large number of adherent cells was observed (Figure 3C). Under high magnification, a small number of cells was found to be round or oval, resembling pebbles. This site was called the pebble area, indicating adherent hematopoietic cells. ADSCs exhibited multilayer growth, the number of adherent cells was significantly increased and ADSCs were found to be converged with each other on the 12th day. Additionally, the cells were significantly larger and had a polygonal or spindle shape (Figure 3D). Transmission electron microscopy showed that ADSCs-Exos were uniform and round, with an evident bilayer structure, typical cup-shaped morphological features, clear edges and low-electron-density material components in the interior. The diameter of ADSCs-Exos varied from 50 to 150 nm (Figure 2). Flow cytometry (FCM) was used to detect specific surface markers of ADSCs: CD44, CD73, and CD105 were present, whereas CD34 and CD45 were absent.

#### 3.2.2 Early adhesion

After 1, 3 and 6 h of culture, the adhesion rate of ADSCs was increased in each group (Figure 3A), indicating that HA-coated porous titanium alloy scaffolds can increase the adhesion of human ADSCs. However, Exos could not promote early adhesion of ADSCs to scaffolds.

**FIGURE 4:**
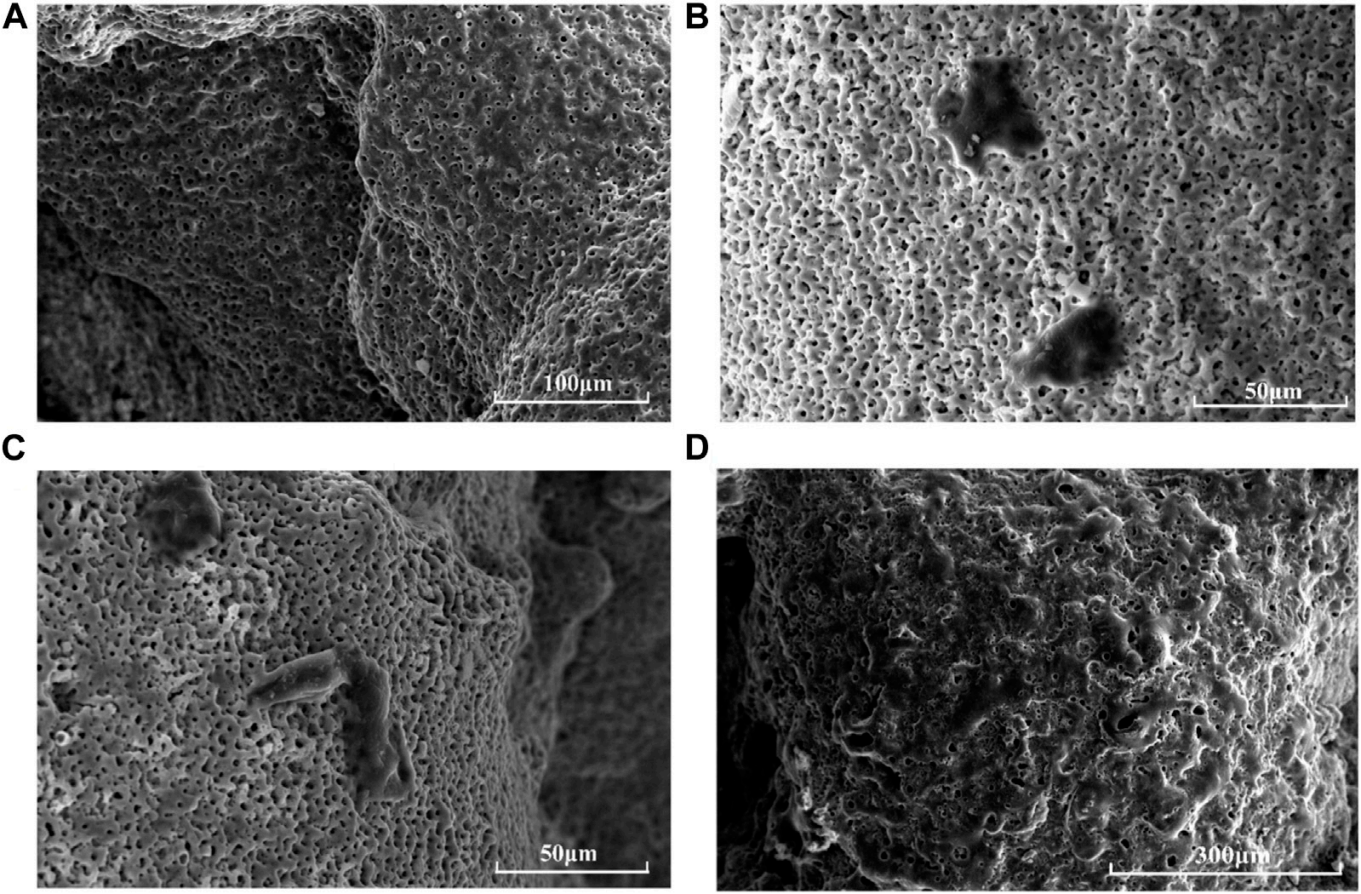
Morphology of ADSCs on HA-scaffolds at different time points by SEM: **(A)** pure scaffolds without adhering ADSCs; **(B)** for 1 day; **(C)** for 3 days; **(D)** for 7 days.

Scattered round or oval ADSCs were found adhered to the scaffold surface on the first day, granular bulges were observed on the surface of ADSCs and a small amount of granular extracellular matrix was found around the cells (Figures 4A,B). Numerous small protrusions were observed on the surface of ADSCs, which were closely bound to the scaffold at the third day. Additionally, the pseudopodia were spread out, showing polygonal and flat protrusions (Figure 4C). The pseudopodia protruding from the cells were significantly elongated and grew across the pore of the scaffold on the seventh day, forming an anchor structure that was firmly bound to the scaffold and connected to the pseudopodia of other cells, and the connected cells resembled a sheet (Figure 4D).

#### 3.2.3 Proliferation of ADSCs in scaffolds

**FIGURE 5:**
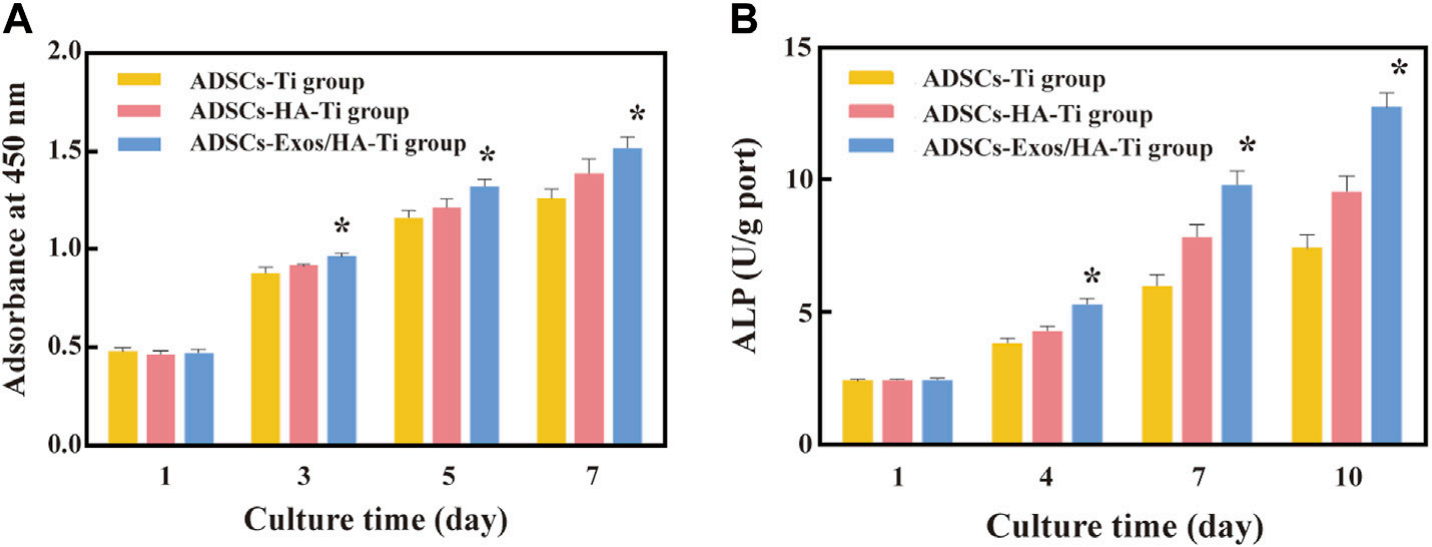
Cytoactive evaluation of ADSCs: **(A)**. Optical density of ADSCs measured by CCK8; **(B)**. ALP test at different detection periods; (*p > 0.05: ADSCs-Exos/HA-Ti *versus* ADSCs-HA-Ti group).

CCK-8 assay was performed to evaluate and compare the proliferation of ADSCs in scaffolds in the ADSCs-Exos/HA-Ti, ADSCs-HA-Ti and control groups (Figure 5A). After 1, 3, 5, and 7 days of culture, the proliferative ability of ADSCs in each group showed an increasing trend. After 1 day of culture, cell proliferation was not significantly different among the three groups (*p* > 0.05). After 3, 5, and 7 days of culture, the number of ADSCs was significantly higher in the ADSCs-Exos/HA-Ti group than in the ADSCs-HA-Ti group (*p* < 0.05). Additionally, the number of ADSCs was significantly higher in these two groups than in the control group (*p* < 0.01). These results indicate that exosomes can promote the proliferation of ADSCs on HA-coated porous titanium alloy scaffolds.

#### 3.2.4 ALP activity

After 1, 4, 7, and 10 days of culture, the ALP activity of ADSCs increased in a time-dependent manner in all three groups (Figure 5B). On the first day, the ALP secretion of each group was low, and gradually increased from the fourth day, reaching the peak levels at the 10th day. No significant difference was observed in ALP activity among the three groups at 1 day (*p* > 0.05). However, ALP activity was higher in the ADSCs-Exos/HA-Ti and ADSCs-HA-Ti groups than in the control group on the 4th, seventh and 10th day(*p* < 0.05). In particular, ALP activity was higher in the ADSCs-Exos/HA-Ti group than in the ADSCs-HA-Ti group.

### 3.3 Results of *in vivo* experiments

#### 3.3.1 Overall observation of rabbit models of mandibular defects

**FIGURE 6:**
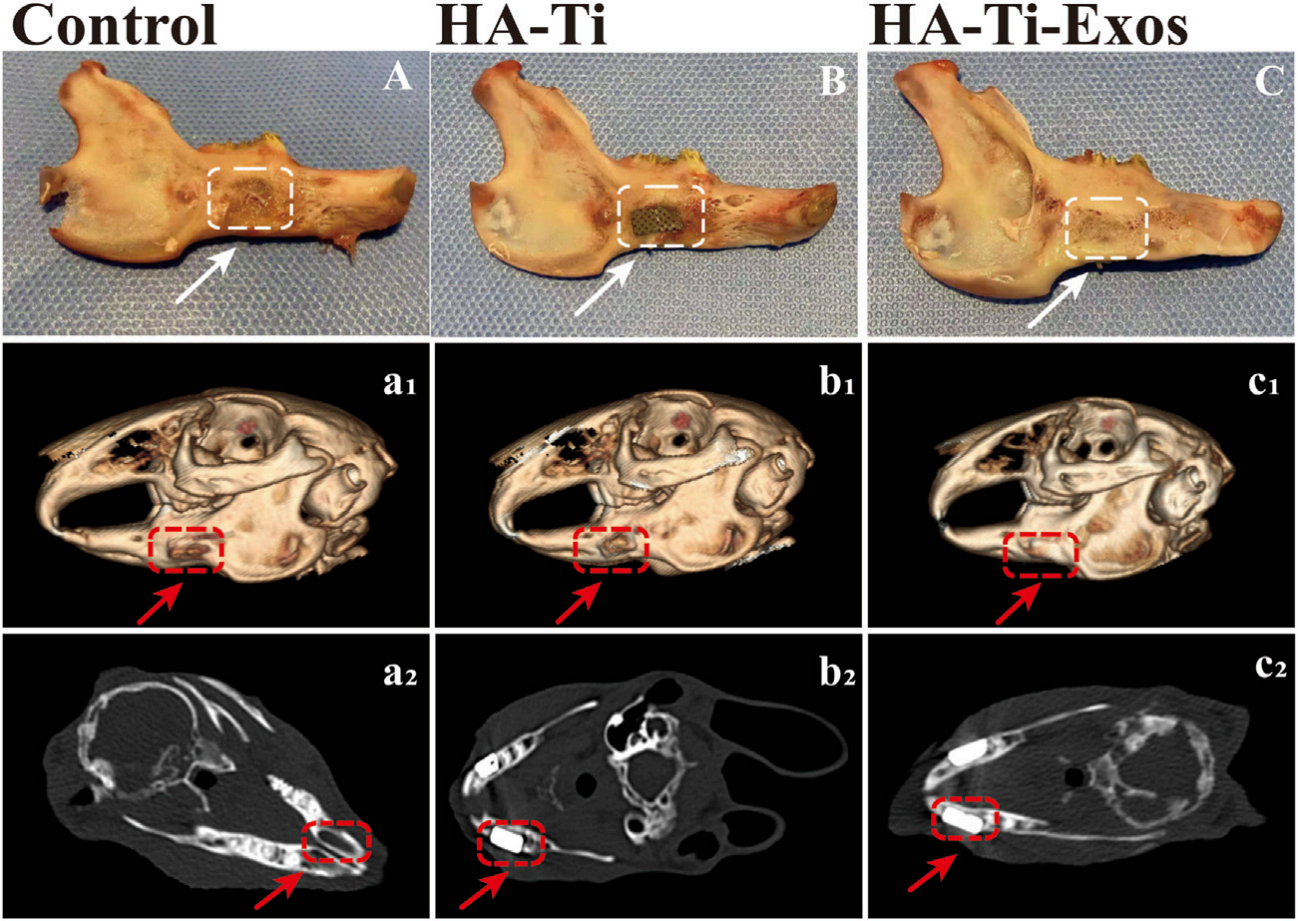
General Observation and Radiological evaluation of a rabbit model of mandibular defects. The dotted lines and arrows indicate the area where the bone defect is located. **(A,A1,A2)** In the control group, no evident new bone formation was observed. **(B,B1,B2)** In the ADSCs-HA-Ti group, new bone was formed but did not completely cover the defect site, and X-ray imaging showed fine low-density shadows around the scaffold. **(C,C1,C2)** In the ADSCs-Exos/HA-Ti group, the defect area was significantly reduced 12 weeks after surgery, and the density of the defect area was similar to that of the original bone tissue.

At 12 weeks postoperatively, the implants in the rabbit-ADSCs-Exos/HA-Ti and rabbit-ADSCs-HA-Ti groups were firmly connected to the edge of the defect site; however, no evident boundary was observed in the rabbit-ADSCs-Exos/HA-Ti group. The surface was partially covered by new bone, which was hard when pressed, but the scaffold materials can still be observed (Figure 6C). In the rabbit-ADSCs-HA-Ti group, the implant was covered by new bone, which was hard when pressed, and the scaffold material could not be seen in the control group (Figure 6B), the bone defect cavity was filled with granulation tissue without significant reduction (Figure 6A).

#### 3.3.2 Imaging analysis

**FIGURE 7:**
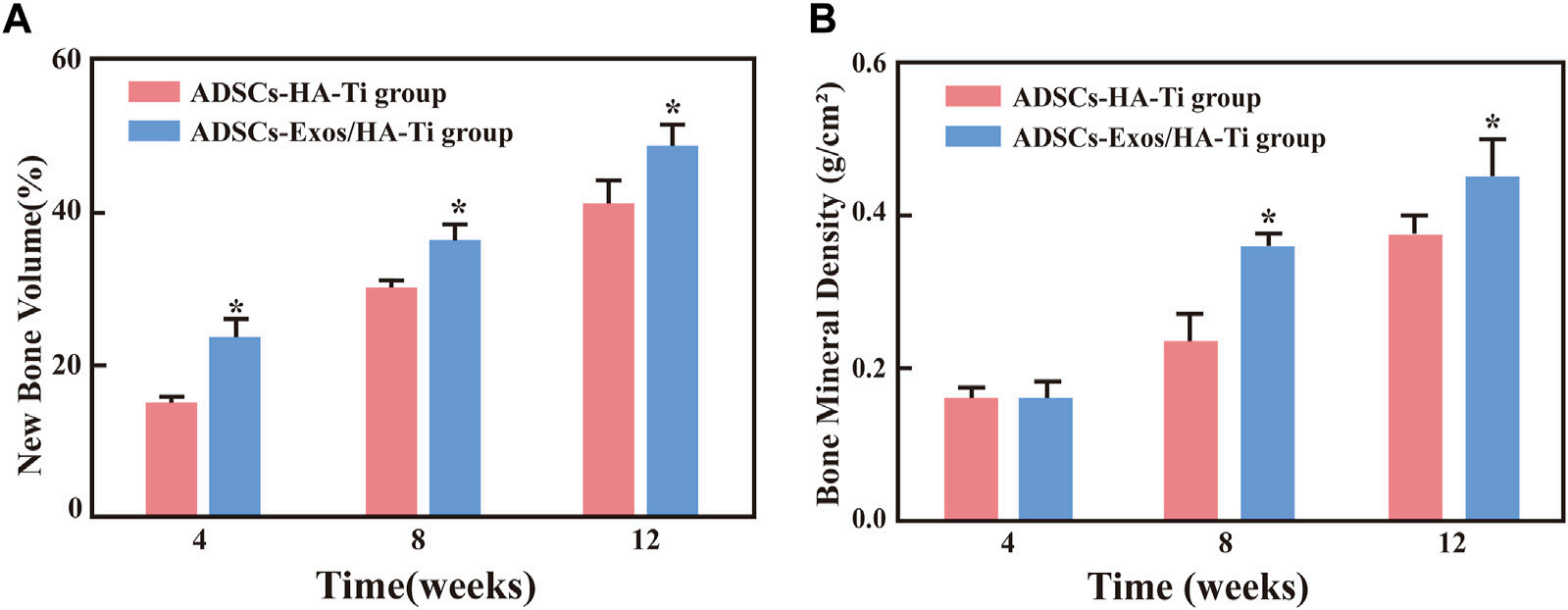
**(A)** The volume fraction of new bone in different groups (*p < 0.05 compared with the ADSCs-HA-Ti group). **(B)** Bone mineral density in different groups (*p < 0.05 compared with the ADSCs-Exos/HA-Ti group).

In the rabbit-ADSCs-Exos/HA-Ti group, Micro-CT images showed that the bone defect disappeared at 12 weeks postoperatively, and X-ray irradiation showed the absence of transmission light and shadow between the scaffold material and bone tissue, indicating that the new bone was closely integrated with the surrounding bone (Figures 6C1, C2). In the rabbit-ADSCs-HA-Ti group, although new bone was formed and the defect area was reduced, the newly formed bone did not cover the entire defect site and was found only on the edge of the defect site. Additionally, X-ray irradiation showed the partial presence of transmission light and shadow between the scaffold material and bone tissue, indicating new bone formation. However, osteogenesis ability was significantly weaker in the rabbit-ADSCs-HA-Ti group than in the rabbit-ADSCs-Exos/HA-Ti group (Figures 6B1, B2). In the blank control group, no new bone formation was observed and the defect area showed no signs of healing even after 12 weeks (Figures 6B1, B2). The newly formed bone was quantitatively assessed using the Micro-CT. After 4 weeks of implantation, the number of newly formed bones was higher in the rabbit-ADSCs-Exos/HA-Ti group than in the rabbit-ADSCs-HA-Ti group, and the rate of new bone formation gradually accelerated during this period. After 12 weeks of implantation, the rabbit-ADSCs-Exos/HA-Ti group had the highest new bone volume (NBV) (Figure 7A).

#### 3.3.3 Evaluation of bone mineral density

The local bone mineral density (BMD) was measured via dual-energy X-ray absorptiometry after 4, 8, and 12 weeks of implantation. BMD increased in the rabbit-ADSCs-Exos/HA-Ti and rabbit-ADSCs-HA-Ti groups in a time-dependent manner (Figure 7B). After 4 weeks, although the BMD of the two groups had increased, no significant difference was observed (*p* > 0.05). However, after 8 and 12 weeks, BMD was higher in the rabbit-ADSCs-Exos/HA-Ti group than in the rabbit-ADSCs-HA-Ti group (*p* < 0.05).

#### 3.3.4 Biomechanical measurement

**FIGURE 8:**
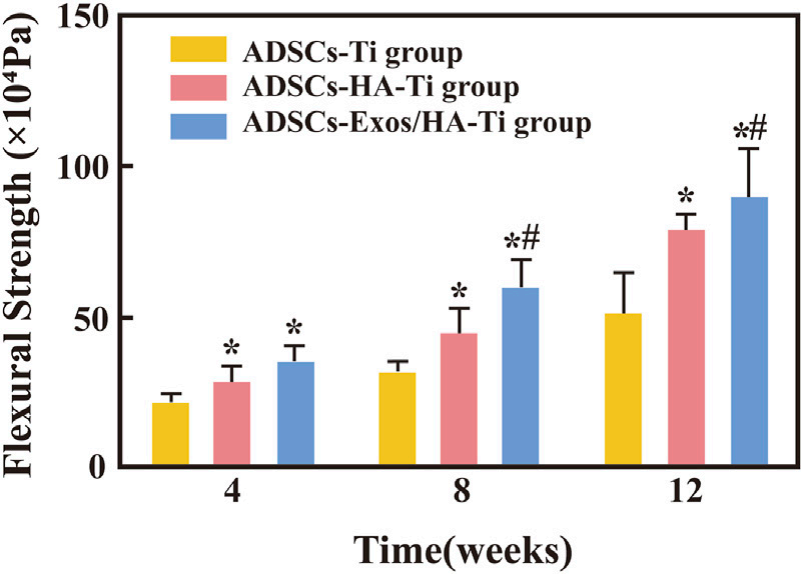
Flexural strength of different groups. (*p < 0.05 compared with the blank control group; #*p* < 0.05 compared with the ADSCs-HA-Ti group.

The bending strength of scaffolds in the three groups was measured after 4, 8, and 12 weeks of implantation. With an increase in implantation time, the flexural strength gradually increased in the three groups, indicating evident bone growth in both types of scaffolds (with and without rabbit-ADSCs-Exos) (Figure 8). After 4 weeks of implantation, the bending strength of the ADSCs-HA/Exos-Ti group and ADSCs-HA-Ti group were different from that of the control group (*p* < 0.05), however, no difference was observed between the ADSCs-HA/Exos-Ti scaffold group and ADSCs-HA-Ti group. After 8 weeks, the bending strength of the ADSCs-HA/Exos-Ti scaffold group and ADSCs-HA-Ti group (59.7 ± 10.096 × 10^4^ Pa and 44.8 ± 8.851 × 10^4^ Pa) was significantly higher than that of the control group (31.25 ± 5.369 × 10^4^ Pa), and a significant difference was observed between the ADSCs-HA/Exos-Ti scaffold group and ADSCs-HA-Ti group (*p* < 0.05). After 12 weeks, the rabbit-ADSCs-Exos/HA-Ti group had the highest flexion strength (89.4 ± 16.801 × 10^4^ Pa).

## 4 Discussion

Our team successfully created a porous scaffold, enriched with calcium and phosphorus, using a combination of hydroxyapatite and titanium through micro-arc oxidation technology. The scaffold’s rough surface is advantageous for the adhesion, proliferation, and expression of osteoblasts (Wehner et al., 2021). The porous titanium alloy scaffold with HA coating meets basic requirements for prostheses, but requires further investigation to ensure histocompatibility for bone tissue engineering (Liu et al., 2020).

Goshima reported that bone marrow stromal cells (BMSCs) injected directly into bone defect sites do not form tissue. However, when incorporated into composite materials and implanted, they promote bone formation (Goshima et al., 1991). Therefore, the combination of scaffold materials and adipose stem cells in this experiment will greatly benefit bone repair. The adhesion of ADSCs to the material surface is one of the key factors for the success of implantation (Sun et al., 2022). It is crucial to assess the biological compatibility of implant materials. The growth of ADSCs on scaffolds is affected by various factors, including the local morphology, surface energy, and chemical energy of the scaffold material (Sun et al., 2022). These surface properties determine how cells will adhere to the surface of the material. By observing the cell morphology and the results of the CCK-8 and ALP, the research revealed that scaffolds made of porous titanium alloy coated with HA have a consistent structure, appropriate pore size, favourable biomechanical characteristics, and excellent biological neutrality.

After adding exosomes, the cell proliferation significantly increased, and the cell activity also increased to some extent. In addition, the results of *in vivo* experiments confirmed that the ideal composite growth factor scaffold should promote osteogenesis, which is beneficial for the formation of new bone, and can serve as a framework to guide the growth of new bone tissue. Exosomes, one of the components of composite scaffolds, play a crucial role in bone tissue engineering and promote osteogenesis. In conclusion, the scaffold developed in this study can promote the proliferation and differentiation of human ADSCs in bone and has a promising osteogenic effect. Exosomes were found to be correlated with the cell proliferation rate. It was observed that the presence of exosomes significantly promoted the survival, proliferation, and differentiation of ADSCs in HA-coated porous titanium alloy scaffolds. Furthermore, the activity of ADSCs varied in composite scaffolds with or without growth factors. This can be attributed to the fact that exosomes can stimulate cell growth and generate new exosomes within the HA-coated porous titanium alloy scaffolds. Exosomes continue to perform biologically and function even after being attached to the scaffolds. On the scaffold material, the ADSCs grew healthily and showed no abnormalities in their cell structure or surface topography. In addition, the cells and the scaffold material had a lot of connections.

Maintaining the biological activity of exosomes is essential for effective bone repair and regeneration in bone tissue engineering (Wang et al., 2020b; Xing et al., 2021). The abovementioned results indicate that exosomes can maintain their activity for a certain period and are released uniformly and continuously into HA-coated porous titanium alloy scaffolds *in vitro*. This discovery could be explained by the following reasons. First, the exposure duration to exosomes was shortened by simultaneously adding exosomes and ADSCs to HA-coated porous titanium alloy scaffolds. Second, in HA-coated porous titanium alloy scaffolds, exosomes can encourage ADSCs to create fresh exosomes and retain their functionality. Exosomes also aided ADSC differentiation and proliferation in a time-dependent manner. Exosomes’ promoting effects on ADSC growth were not immediately apparent but became clear after 3 days of culture. Exosomes in HA-coated porous titanium alloy scaffolds continued to be biologically active for 2 weeks. However, the interaction between ADSCs and the scaffold material should be further investigated to determine the application prospects of HA-coated porous titanium alloy scaffolds in bone tissue engineering.

Exosomes and hydroxyapatite have been widely studied for their good osteogenic properties. It was found that they can combine efficiently and have good bone affinity, providing biocompatible substrates for cell adhesion, survival, proliferation and osteogenic potential (Yang et al., 2020; Lee et al., 2023). There may be some chemical bonding between exosomes and hydroxyapatite, but there are few reports on this, and future research on this aspect should make the development of bone tissue engineering a higher level. The results of the experiment indicate that while exosomes may not have a significant impact on the initial adhesion of adipose stem cells, they do increase cell proliferation and activity. Furthermore, other studies have shown that exosomes can promote cell growth on the surface of titanium and that there is a positive correlation between cell adhesion and proliferation on different biomaterial substrates (Das et al., 2007; Wang et al., 2020a). One of the important factors for successful material implantation is the adhesion ability of ADSCs acting as anchor cells on the surface of materials. The adhesion of cells on the surface of materials and the formation of good cell morphology are important indicators in evaluating the biocompatibility of implant materials. Marklein et al. (2016) found that cell morphology can also be used as a predictor of progenitor cell fate. This beneficial effect is facilitated by exosome-mediated factor communication, which leads to cytoskeletal rearrangement, adhesion, diffusion, and differentiation. It is not yet clear how exosomes affect cellular behaviours, although they are believed to mediate receptor cell regulation through the transfer of exosomes cargo, such as mRNA and microRNA (Valadi et al., 2007), other possible mechanisms include exosome-mediated molecular signalling between cells and the promotion of cytoskeletal remodelling and adhesion junction formation. Additionally, determining the optimal concentration of exosomes for osteogenic effects remains a challenge for future research (Lan et al., 2020).

Therefore, scaffolds based on porous titanium alloys with HA composite coating are an excellent substitute for bone transplantation and have strong research and application value. Subsequent *in vivo* experiments showed that HA coating was not easily degraded in tissues and body fluids and promoted bone integration to the scaffold. The addition of exosomes significantly improved the biomechanical properties of the implant-bone interface.

In the present study, the influence of composite materials as bone implants on bone tissue repair and bone union was evaluated through *in vitro* and *in vivo* experiments. The combined application of bone tissue engineering and stem cell therapy provided the theoretical basis and experimental basis for clinical development and application of new materials for bone defect repair. However, whether composite materials can be used in clinics needs further study.

## 5 Conclusion

The porous titanium alloy was prepared by SLM technology, and HA bioactive coating was constructed on the metal trabecular surface of porous titanium alloy by MAO, which combined the high mechanical strength of metal materials with the bone induction ability of bioactive ceramics. The titanium composite exhibits excellent properties, like porosity of about 69%, elasticity modulus of about 3.2 GPa and compressive strength of about 92 MPa. *In vitro* co-culture experiments proved that HA-coated porous titanium scaffolds have favourable biocompatibility and low toxicity for the growth, proliferation and differentiation of ADSCs. And adipose-derived exosomes significantly promoted the growth of ADSCs in HA-coated porous titanium scaffolds. *In vivo* experiments demonstrated that exosomes extracted from rabbit ADSCs can promote the growth of ADSCs and osteogenesis, and the ADSCs-Exos/HA-Ti scaffolds have been successfully used to repair the rabbit mandibular defects. In the present study, the biocompatibility of ADSCs-Exos/HA-Ti scaffolds as bone tissue engineering scaffolds were evaluated, providing a theoretical and experimental basis for the design of such scaffolds and laying a solid foundation for promoting the clinical application of stem cells combined with bone tissue engineering. Although this study demonstrated the synergistic positive effects of HA-Ti scaffolds and ADSCs-Exos on bone tissue regeneration, the underlying mechanism warrants further investigation, which is also the direction of our future research.

## Data Availability

The original contributions presented in the study are included in the article/Supplementary Material, further inquiries can be directed to the corresponding authors.
